# Point-of-care platelet function testing for guided transfusion in neurosurgical management of intracranial hemorrhage: a systematic review

**DOI:** 10.1186/s40001-022-00819-4

**Published:** 2022-10-01

**Authors:** Flora Wen Xin Xu, Nicole-Ann Lim, Ming Ann Sim, Lyn Li Lean, Ne-Hooi Will Loh, Ka Ting Ng, Vanessa Tze Yuh Chua, Sophia Tsong Huey Chew, Lian Kah Ti

**Affiliations:** 1 Yong Loo Lin School of Medicine, National University of Singapore, Singapore, Singapore; 2 Department of Anesthesia, Ng Teng Fong General Hospital, Singapore, Singapore; 3 Department of Anesthesiology, Faculty of Medicine, University of Malaya, Kuala Lumpur, Malaysia; 4 Department of Anesthesia, Singapore General Hospital, Singapore, Singapore; 5 Department of Anesthesia, National University Hospital, Singapore, Singapore; 6 Department of Anesthesia, Yong Loo Lin School of Medicine, National University of Singapore, Singapore, Singapore

**Keywords:** Point-of-care test, Platelet function, Neurosurgery, Perioperative care

## Abstract

Given the rising prevalence of antiplatelet therapy, rapid preoperative identification of patients with bleeding diathesis is necessary for the guidance of blood product administration. This is especially relevant in neurosurgery for intracranial hemorrhage (ICH), where indiscriminate transfusions may lead to further hemorrhagic or thromboembolic injury. Point-of-care (POC) testing of platelet function is a promising solution to this dilemma, as it has been proven effective in cardiac surgery. However, to date, POC platelet function testing in neurosurgery has not been extensively evaluated. This systematic review appraises the use of POC platelet function test (PFT) in emergency neurosurgery in terms of its impact on patient outcomes.

A comprehensive search was conducted on four electronic databases (Pubmed, MEDLINE, Embase, and Cochrane) for relevant English language articles from their respective inceptions until 1 June 2022. We included all randomized controlled trials and cohort studies that met the following inclusion criteria: (i) involved adult patients undergoing neurosurgery for ICH; (ii) evaluated platelet function via POC PFT; (iii) reported a change in perioperative blood loss; and/or (iv) reported data on treatment-related adverse events and mortality. Assessment of study quality was conducted using the Newcastle Ottawa Quality Assessment Scale for Cohort Studies and Case–Control Studies, and the JBI Critical Appraisal Checklist for Case Series.

The search yielded 2,835 studies, of which seven observational studies comprising 849 patients met the inclusion criteria for this review. Overall, there is evidence that the use of POC PFT to assess bleeding risk reduced bleeding events, thromboembolic adverse outcomes, and the length of hospitalization. However, there is currently insufficient evidence to suggest that using POC PFT improves blood product use, functional outcomes or mortality.

**Supplementary Information:**

The online version contains supplementary material available at 10.1186/s40001-022-00819-4.

## Introduction

Neurosurgical intervention for intracranial hemorrhage (ICH) typically carries a poor prognosis, with 1 year mortality rates of up to 59.0% at 1 year post-hemorrhage [1]. In-hospital mortality rates remain high at 41.6% [2] despite surgical intervention with decompressive craniotomies. One significant cause of poor morbidity and mortality among patients with ICH would be the use of antiplatelets, which is associated with increased hematoma volume growth [3]. However, blood product transfusion guidelines for management of such complications vary across institutions. Targeted transfusion remains the last frontier in effective surgical management of ICH, and we posit that perioperative assessment of bleeding risk using a POC PFT is a useful means to guide the transfusion of blood products.

There are several methods to assess platelet function. A group of assays, collectively known as PFTs, uses specialized instruments to measure the ability of platelets to aggregate and promote clotting in a sample of blood. There are a variety of such tests available, but there is no one test that can identify all problems with platelet function. In addition, there is no widespread agreement on which test is best for each circumstance. The gold standard light transmission platelet aggregation test (LTA) is time-consuming and less suitable for rapid decision-making in emergency neurosurgery [4]. In contrast, POC PFTs have a faster turnaround time than certain standard coagulation tests (SCTs), making them the optimal choice for the guidance of platelet transfusions in neurosurgical emergencies. Common POC platelet function testing methods include thromboelastography (TEG) (TEG® 5000/6 s Hemostasis Analyzer System), rotational thromboelastography (ROTEM) (ROTEM® Delta System), platelet reactivity turbidimetry test (VerifyNow® System), multiple electrode platelet aggregometry (Multiplate® Analyzer) and platelet function analyzer (PFA®) (PFA®-100/200).

In current clinical practice, physicians depend heavily on SCTs to guide transfusion. Common indices of platelet function used in SCTs include activated partial thromboplastin time (aPTT), platelet count, and prothrombin time (PT), from which the international normalized ratio (INR) is derived after adjustment for the potency of the thromboplastin reagent used. These tests are useful in the emergency setting because of the quick turnaround time of the tests, but they confer limited information on coagulation status [5, 6] and do not accurately predict bleeding risk in coagulopathic patients [6–8]. While SCTs reflect quantitative information, such as platelet cell count, they do not offer valuable insight into qualitative factors, such as platelet function.

Moreover, the rising prevalence of antiplatelet therapy poses a significant challenge to accurate assessment of platelet function for guided transfusion. Patient response to antiplatelets has proven heterogenous, and there is a growing population of antiplatelet resistant patients who present with uninhibited platelet function at the point of transfusion [9]. Overtransfusion of blood products puts such patients at unnecessary risk of immunologic and non-immunologic adverse reactions. Nonetheless, patients on antiplatelet therapy are often transfused with platelets prophylactically to correct bleeding diathesis [10], regardless of platelet count or function.

Unguided transfusion presents significant problems. First, blood product transfusions may not avert rebleeding in all patients [11–13], due to varied platelet responsiveness and patient comorbidities. Second, blood product transfusion is a major risk factor for the development of neurosurgical complications, such as bleeding [14], neurological deterioration [15] and reoperation [15] within 30 days [14]. These patients also had greater odds of dying or having poorer functional outcomes [15].

POC PFT has been shown to be effective in reducing blood transfusion in cardiac surgery [16] and in predicting the risk of perioperative blood loss in high risk cardiac surgery patients [17].

Therefore, there is a need to evaluate the use of POC PFT in guiding blood product transfusions in neurosurgical patients, particularly those on antiplatelet therapy. In this review, we examined the clinical use of POC PFT in the guidance of blood product transfusions in the neurosurgical management of ICH and assessed their impacts on patient outcomes.

## Methods

**Fig. 1:**
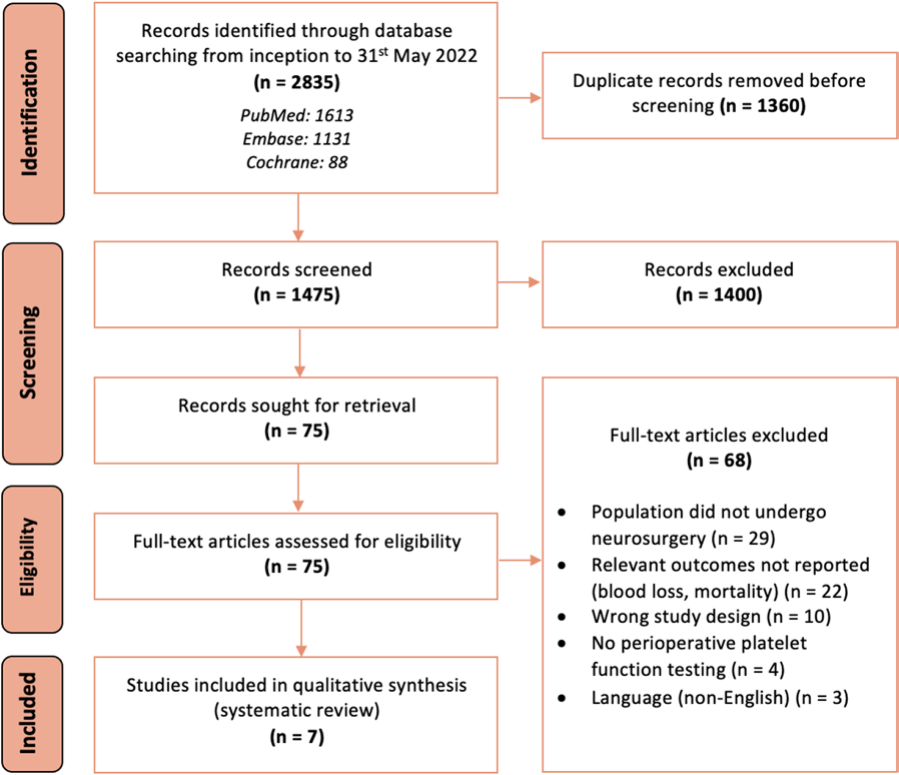
PRISMA diagram

This review was conducted according to the Preferred Reporting Items for Systematic Reviews and Meta-Analyses (PRISMA) guidelines as shown in Fig. 1. The review protocol was registered with PROSPERO (CRD42020213180).

### Eligibility and search strategy

We systematically searched Pubmed, MEDLINE, EMBASE and Cochrane databases from their respective inceptions till 1 June 2022. The search keywords included a combination of “point of care”, “platelet function”, “thromboelastography”, “intracranial hemorrhage” and “neurosurgery”. We included all adult patients who have undergone a perioperative POC PFT for neurosurgical intervention following cranial bleeds. A hand search was conducted for gray literature and the bibliographies of included studies.

### Study selection

We included all randomized controlled trials, observational studies and case series that met the following inclusion criteria: (i) adult patients who underwent neurosurgery, (ii) indication for neurosurgery was any form of ICH; (iii) evaluated platelet function via POC PFT; (iv) reported a change in perioperative blood loss; and/or (v) reported data on treatment-related adverse events and mortality.

We excluded studies that did not compare differences in outcomes between patients with and without POC PFT. Animal studies and pediatric studies were excluded. We also excluded case reports, review articles, editorials, and guidelines. Two authors (FWXX, NSL) independently performed the initial screening of the titles and abstracts identified in the primary search, followed by a full-text review of relevant studies. Any discrepancy in the article selection was resolved by consensus between FWXX and NSL, with the help of a senior author (LLL), if necessary.

### Data extraction

The data from each study were independently extracted by two authors (FWXX, NSL) from the main text and supplementary files of included studies. Apart from the measured outcomes, other data included study design, sample size, and participant demographics. Due to the limited number of studies and heterogenous outcome data available, data pooling for meta-analysis was not performed. A narrative synthesis of the data was performed to explore the role of POC PFTs in guiding perioperative platelet transfusion of neurosurgical candidates following ICH. Our primary outcome was the frequency of perioperative transfusion of blood products, such as packed red blood cells (pRBC), platelets, fresh frozen plasma (FFP), and prothrombin complex concentrate (PCC). Our secondary outcomes were postoperative bleeding, thromboembolic complications, functional outcomes, length of hospitalization, and mortality.

### Quality assessment

Assessment of study quality was conducted using the Newcastle Ottawa Quality Assessment Scale[18] for Cohort Studies and Case–Control Studies. Case series were evaluated using the JBI Critical Appraisal Checklist for Case Series [19] (Additional file 1 Tables S1 and S2).

## Results

### Search results and population characteristics

Our search yielded 2,835 studies which we screened for relevance based on the title and abstract. After excluding irrelevant studies and removing duplicates, we reviewed the full text of the 75 remaining studies. Another 68 studies were then excluded, for the following reasons: (i) the patient population did not undergo neurosurgery for ICH (*n* = 29); (ii) blood loss or mortality outcomes were not reported as outcomes (*n* = 22); (iii) study design was a case report, case series or review (*n* = 10); (iv) patients did not undergo platelet function testing (*n* = 4); and (v) the report was not in the English language (*n* = 3). Finally, seven studies that fulfilled the inclusion criteria were analyzed for this review. Figure 1 depicts the PRISMA flowchart.

### Characteristics and quality of studies

Among the included studies, four were single-arm studies examining neurosurgical patients who have undergone a POC PFT, whereas three were double-arm studies comparing the use of POC PFT and SCT. Three of the studies dealt with neurosurgical clipping and coiling for aneurysms [20–22], two involved decompressive craniotomies [23, 24], and one involved external ventricular drain insertion [25]. One study involved a wide range of emergent neurosurgical procedures ranging from craniotomies to hematoma evacuation [26].

Four studies (Li [22], Ellenberger [24], Vahtera [20] and Rimaitis [23]) used either TEG or ROTEM. Von der Brelie et al. [21] utilized the PFA-100®. Beynon and team [26] used the Multiplate® Analyzer. Majmundar et al. [25] used the VerifyNow® Assay. Baseline characteristics of the seven included studies are summarized in Table 1.

**Table 1:** Baseline characteristics of 7 included studies

Paper	Year	Country	Study type	Platelet function test	Cohort size (case/control)	Type of neurosurgery
Beynon	2013	Germany	Prospective cohort	Multiplate	21	Craniotomy (33.3%) burr hole trephination (19.0%) EVD insertion (9.5%) None (14.2%)
Ellenberger	2017	Switzerland	Prospective cohort	ROTEM	92	Emergency neurosurgery for SAH and/or SDH (68.5%) decompressive craniectomy (26.1%)
Li	2021	China	Retrospective cohort	TEG	145 (93/52)	Stent assisted coiling
Majmundar	2019	USA	Retrospective cohort	VerifyNow	345 (208/137)	EVD insertion
Rimaitis	2020	Lithuania	Prospective cohort	ROTEM	134 (69/65)	Craniotomy
Vahtera	2019	Finland	Prospective cohort	ROTEM	33 (17/16)	Occlusion of the ruptured aneurysm by either endovascular coiling or surgical clipping
Von der Brelie	2018	Germany	Retrospective cohort	Platelet function analyser	79	Occlusion of the ruptured aneurysm by either endovascular coiling or surgical clipping

ROTEM parameters reported were EXTEM, INTEM, FIBTEM and APTEM. EXTEM, an extrinsically activated assay with recombinant tissue factor, measured coagulation mediated by the extrinsic pathway. INTEM, an intrinsically activated assay using phospholipid–ellagic acid, measured clot formation via the contact phase. FIBTEM measured the formation of fibrin-based clots after platelet inhibition by cytochalasin D, a glycoprotein IIb/IIIa receptor blocker. APTEM, an aprotinin-based test, detects ongoing hyperfibrinolysis. Other measured variables reported include: clotting time (CT), which is the time from the start of the sample until the amplitude of clot formation reaches 2 mm (R, min); clot formation time (CFT), which is the time elapsed from R until a 20 mm amplitude is achieved (K, min); time to reach maximum speed of initial clot formation (α angle, deg); maximum clot firmness (MCF, mm); maximum clot strength (MA, mm); clot amplitude after 15 min (CA15); percent of clot lysis 30 min after MA (EPL); and percent of amplitude decay 30 min after MA (LY30). Overall, a hypercoagulable state can be detected if the MCF is elevated. Using different reagents, the effect of platelets and fibrinogen (EXTEM-MCF) and fibrin formation and its polymerization (FIBTEM-MCF) on clots can be distinguished.

### Blood product use

Six studies [20, 21, 23–26] reported the use of blood products, 2 [23, 25] of which compared transfusion between two patient groups—a group that underwent POC PFT and another group that underwent SCT instead. Results are summarized in Table 2. Rimaitis et al. [23] found no significant difference in the percentage of patients requiring transfusion between the two groups (35.4% vs. 34.8%). For transfusion of pRBCs in particular, a significantly larger proportion of patients were transfused in the TEG-tested group than in those who only underwent SCT (21.7% vs. 15.4%). Majmundar et al. [25] reported significantly higher rates of pRBC transfusion in patients who underwent the VerifyNow® assay than in patients who did not (34.6% vs. 13.9%, *p* < 0.0001). Among the patients who had inhibited platelet function as evidenced by the VerifyNow® Assay, there was no significant difference in the incidence of postoperative ICH between transfused and non-transfused groups (12.2% vs. 18.2%, *p* = 0.4557).

**Table 2:** Transfusion products and hemostatic agents administered

Paper	Blood products	Prevalence/Volume of Transfusion
Beynon	Platelet concentrate	28.6% overall
	Tranexamic acid	28.6% overall
	Desmopressin	28.6% overall
Majmundar	Platelets	VerifyNow 72% Control 19%
Rimaitis	RBCs	ROTEM 18.8% Control 15.4%
	Fresh frozen plasma	ROTEM 13% Control 16.9%
	Platelet	ROTEM 11.6% Control 10.8%
	Prothrombin complex concentrate	ROTEM 36.2% Control 38.5%
	Cryoprecipitate	ROTEM 10.1% Control 3.1%
	Tranexamic acid	ROTEM 36.2% Control 38.5%
Vahtera	Tranexamic acid	3% overall
Von der Brelie	Blood	2.7 units overall
Ellenberger	RBC	High bleeders 5 units Low bleeders 1 units
	Fresh frozen plasma	High bleeders 3 units Low bleeders 2 units
	Platelets	High bleeders 2 units Low bleeders 2 units
	Prothrombin complex concentrate	High bleeders 1.8 × 10^3 units Low bleeders 3 × 10^3 units
	Fibrinogen	High bleeders 4 g Low bleeders 4 g
	Recombinant Factor VII	High bleeders 7 mg Low bleeders 0 mg
	Tranexamic acid	High bleeders 1.5 g Low bleeders 1.0 g

Ellenberger [24] stratified outcomes in terms of high bleeders (receiving ≥ 3 pRBCs or requiring reoperation for hematoma drainage) and low bleeders (< 3 pRBCs and not requiring reoperation for hemostasis). Hemostatic therapy included transfusion of FFP, PCCs, platelet concentrates, activated factor VII and tranexamic acid (TXA), guided by ROTEM and clinical judgment. High bleeders received a median of 5 units pRBC, 3 units FFP, 2 units platelet concentrates, 1.8 units PCCs, and 1.5 units TXA, while low bleeders received 1 unit pRBC, 2 units FFP, 2 units platelet concentrates, 3 units PCCs and 1 unit TXA. Overall, the following ROTEM parameters were found to be the best predictors of high bleeders: MCF-EXTEM (AUC 0.72, 95% CI 0.61–0.83), MCF-FIBTEM (AUC 0.71, 95% CI 0.60–0.82), INTEM-MCF (AUC 0.70, 95% CI 0.59–0.81) and fibrinogen levels (AUC 0.70, 95%CI 0.58–0.82).

In the two studies (Rimaitis et al., Majundar et al.) comparing POC PFT cohorts with SCT cohorts, a significantly higher percentage of patients in the POC PFT cohorts underwent transfusion. It was unclear if the total volume of blood products transfused was higher for patients who have undergone a POC PFT. The paucity of data comparing POC PFT with standard testing calls for more primary research studies to be conducted on the topic, as POC PFT is a novel approach in neurosurgical management.

### Bleeding events

Six of the studies [21–26] reported bleeding events (Table 3). Among the studies that compared POC PFT to SCT, 2 studies [22, 23] reported a significant benefit when using POC PFT to predict bleeding and guide transfusion requirements, but 1 [25] did not.

**Table 3:** Postoperative complications and survival

Paper	Subgroup	*n*	Bleeding complications (%)	Thromboembolic complications (%)	Favorable functional outcomes (%)	Length of hospitalization (days)	Mortality (%)
Beynon	Overall	21	1 (4.8%)	0	–	–	1 (4.8%)
Ellenberger	High bleeders	39	–	–	–	31 (79.5%)	14 (35.9%)
	Low bleeders	53	–	–	–	23.5 (44.3%)	11 (20.8%)
Li	TEG	93	5 (5.38%)	21 (22.6%)	89 (95.7%)	–	–
	Control	52	6 (11.5%)	15 (28.8%)	50 (96.2%)	–	–
Majmundar	VerifyNow	208	39 (18.8%)	–	–	–	–
	Control	137	22 (16.1%)	–	–	–	–
Rimaitis	ROTEM	69	31 (44.9%)	–	32 (46.4%)	17 (24.6%)	18 (26.1%)
	Control	65	21 32.3%)		23 (35.4%)	22 (33.8%)	2 (3.1%)
Vahtera	ROTEM	17	–	–	14 (82.4%)	–	1 (5.9%)
	Control	16	–	–	–	–	–
Von der Brelie	Impaired platelet function	55	5 (9.0%)	–	17 (30.9%)	–	17 (21.5%)
	Normal platelet function	24	2 (8.3%)	–	8 (33.3%)	–	

In the study by Rimaitis et al.[23] (*n* = 134), patients with severe traumatic brain injury undergoing emergent craniotomies had their intraoperative coagulation management guided by either TEG or SCT. In the TEG-guided group, there was a significantly lower incidence of progressive hemorrhagic injury as compared with the control group that underwent SCT (30.4% vs. 47.7%, *p* = 0.04). Subgroup analysis based on the existing coagulopathy status of patients showed the TEG-guided group had a lower need for neurosurgical reintervention as compared with the control group that only underwent SCT (12.9% vs. 40%, *p* = 0.020). Estimated intraoperative blood loss reported did not differ between the groups (400 mL for both).

Li et al. [22] (*n* = 145) reported similar results, with a significantly lower risk of minor bleeding events in patients managed with TEG than in those managed with SCT (please insert data, with P value). Minor bleeding events were defined as extracranial bleeding that did not cause clinical deterioration.

In contrast, Majmundar et al.[25] (*n* = 345) reported that the use of VerifyNow® assay intraoperatively during extraventricular drain placement did not significantly affect the incidence of catheter-induced hemorrhage. However, the mean age of those receiving VerifyNow® Assay was significantly higher than the control patients (54.5 vs. 38.5 years, *p* < 0.0001). The significant age difference may confound outcomes and contribute to the heterogeneity of findings. There were also no institutionalized guidelines on when the VerifyNow® assay should be used, with the decision being left to clinical judgment.

Overall, two of three studies comparing POC PFT with SCT found that the use of POC PFT significantly reduced the risk of postoperative bleeding events and neurosurgical reintervention.

### Thromboembolic adverse outcomes

Two studies [22, 26] reported thrombotic or embolic outcomes (Table 3), which may occur when there is an excessive correction of bleeding diathesis. One study reported no thromboembolic event [26]. In the study by Li et al. [22], proportionately fewer patients in the TEG-tested group reported minor thromboembolic adverse events compared with the SCT group, but results were not statistically significant (21.5% vs. 28.8%; *p* = 0.41). Rates of major thromboembolic events reported in the TEG and SCT groups were comparable (1% vs. 0%; *p* = 1.00). Minor events were defined as new asymptomatic infarctions diagnosed by diffusion-weighted imaging, while major events were defined as newly developed transient ischemic attacks or symptomatic ischemic infarctions. Overall, TEG-tested patients appear to have a lower rate of minor thromboembolic adverse events, although this did not reach statistical significance.

### Functional outcomes

Four studies reported functional outcomes at discharge [21–23] and at 90 days [20] (Table 3). Three studies [20] reported their outcomes in terms of the modified or extended Glasgow Outcome Scale, while one study used the modified Rankin scale [22]. Two studies [22, 23] reported functional outcomes.

The overall prevalence of good functional outcomes was 41.7% at discharge in the study by Von der Brelie [21] and 58.8% at 90 days in the study by Vahtera [20]. When comparing POC PFT with control, both Li et al. and Rimaitis et al. [23] found there was no significant difference in the rates of favorable functional outcomes.

### Length of hospitalization

Ellenberger [24] noted a significantly longer average length of hospital stay for high bleeders requiring transfusion of ≥ 3 pRBCs compared with low bleeders (31 days vs. 23.5 days), as well as longer ICU stay in the high bleeder group (10 days vs. 5.5 days). Findings are summarized in Table 3.

Rimaitis [23] reported a significantly reduced length of hospitalization in coagulopathic patients who underwent ROTEM, compared with those who only underwent SCT (19 days vs. 25 days). Moreover, lower rates of surgical reintervention were indicated in ROTEM-guided hemostatic therapy (10/65; 15.4%) than in the SCT-guided (4/69; 5.80%).

### Mortality

All of the single-arm [20, 21, 24, 26] and one of the double-arm studies[23] reported the incidence of mortality (Table 3). In the study by Rimaitis [23], there was no significant difference (*p* = 0.686) in mortality outcomes between TEG and SCT groups. Ellenberger [24] corroborated that there was no significant difference between high bleeders (received ≥ 3 pRBCs, or required reoperation for hematoma drainage) and low bleeders (received < 3 pRBCs and not requiring reoperation for hemostasis) (35.9% vs. 20.8%; 14/39 vs. 11/53; *p* = 0.107). In Vahtera’s study [20], one patient (5.9%; 1/17) died. In Von der Brelie’s study [21], in-hospital mortality was 21.5% (17/55). In the study by Beynon [26], one patient died (4.76%; 1/21) due to postoperative rebleeding. Overall, there was insufficient evidence to suggest an association between POC PFT use and mortality benefit.

## Discussion

Our review found that the use of POC PFTs such as ROTEM or TEG correlated with a significant reduction in perioperative blood loss, postoperative hemorrhagic and thromboembolic complications, as well as rates of surgical reintervention and length of hospitalization [23, 24]. However, there remains insufficient evidence to show that the use of POC PFT improves blood product use, functional outcomes, or mortality. Certain parameters such as MCF outperformed others in predicting a need for blood transfusion. On the contrary, PFA, Multiplate® Analyzer and VerifyNow® Assay were used less frequently and outcomes reported were heterogeneous.

To our knowledge, this is the first systematic review conducted on the use of POC PFT in this neurosurgical population. Consistent with existing literature on POC testing of platelet function in cardiac surgeries[27] and spine surgeries [28], this review shows POC testing of platelet function has the potential to improve patient outcomes such as mortality when used perioperatively. The European Association of Cardiothoracic Anaesthesiology (EACTA) recommends that POC testing of platelet function be considered for neurosurgical patients on dual antiplatelet therapy or P2Y12 receptor inhibitors [29]. Our results are concordant with a recent meta-analysis on the role of POC PFT in cardiac surgery [27], which also shows a reduction in postoperative bleeding events with the use of POC PFT. Therefore, a POC PFT is likely sufficient to guide the use of blood products for the correction of bleeding diathesis in neurosurgery for ICH.

In contrast with existing literature [27, 28], we found that POC testing of platelet function led to increased blood product transfusion, particularly pRBC, in the neurosurgical population studied. This may be due to a discrepancy in the institutional or physician-specific threshold for blood product transfusion in the neurosurgical patients, as there is still no consensus on the superiority of either a restrictive or liberal transfusion protocol [30]. While increased blood product use may raise concerns of over-transfusion, with its accompanying increased risk of mortality [31], thromboembolic and ischemic complications [32], our review found that POC PFT-guided hemostasis management of patients resulted in a lower risk of thromboembolic complications. This suggests that POC PFT may accurately guide therapeutic dosing of blood product use. However, there is still limited data to show that POC platelet function testing improves overall patient outcomes. Only one study [23] demonstrated a significant reduction in length of hospitalization among neurosurgical patients, but there was no significant benefit in terms of functional outcomes [22, 23] and mortality [23].

The choice of POC PFT and testing protocol has to be adapted to each institution’s needs, as they each have their advantages and detriments. While LTA is the historical gold standard for platelet function testing, it requires high sample volumes, manual sample processing and long processing times [4]. POC testing most commonly involves the use of ROTEM and TEG in current practice. Both are global hemostasis tests that assess platelet function and coagulation profile by assaying similar parameters of clot formation dynamically in whole blood. Newly modified TEG systems with platelet mapping (TEG-PM) now account for use of aspirin (TEG-PM-AA, arachidonic acid) and thienopyridines (TEG-PM-ADP, adenosine-diphosphate). Benefits of ROTEM and TEG use include improved prediction of bleeding, reduced need for blood transfusions and improved clinical outcomes [4].

One study involving trauma patients showed that TEG-PM-AA, Multiplate® Analyzer, and VerifyNow® Assay were also able to identify antiplatelet use [33], which could make them useful for guiding hemostatic management in patients without clear drug history. The VerifyNow® Assay can identify patients with normal platelet activity who are on antiplatelet agents, thereby reducing the risk of thrombotic event [34]. However, it is expensive and inflexible, with limited hematocrit count [4]. PFA is a good screening tool for platelet dysfunction due to its high negative predictive value and ease of use [35]. However, as it generally assesses primary hemostasis, it is not possible to identify which aspect of primary hemostasis is affected [36]. Therefore, it may be less useful for clinicians hoping to identify and correct platelet dysfunction. The Multiplate® Analyzer is a rapid screening tool that requires little technical training to use [4]. Numerous studies in cardiac surgery have proved that it may accurately predict postoperative bleeding risk [37, 38]. However, like PFA, it cannot identify mild platelet dysfunction [39].

Overall, ROTEM and TEG are the most widely adopted POC PFT modalities, particularly in management algorithms for surgical and emergency settings. They have been validated as high-fidelity tests that offer reliable predictive evaluation of the risk of increased postoperative bleeding and blood product use.[4].

## Limitations

While this review conducted an extensive and robust search among numerous databases and the gray literature, there was a paucity of randomized controlled trials or controlled observational studies on this emerging topic. Included studies were largely heterogeneous in terms of the types of neurosurgery undergone by ICH patients and outcomes reported. The quality of included studies were variable, as study participants often underwent POC PFT based on each neurosurgeon’s discretion, leading to non-consecutive and clinician-dependent recruitment of participants. Moreover, the majority of the studies did not report transfusion volumes and thresholds. As a result, blood loss outcomes could not be conclusively compared between studies. Nonetheless, these variances appear to be a reflection of the variances in clinical practice, especially as guidelines in perioperative management of neurosurgery for ICH are ever evolving.

## Conclusions

There exists early evidence for the use of POC PFT for guided hemostatic therapy in neurosurgical emergencies. Our systematic review found that POC testing of platelet function in emergency neurosurgeries for ICH may reduce bleeding events and thromboembolic adverse outcomes, and shorten the length of hospitalization. TEG and ROTEM are the most commonly adopted modalities in our study, and are reported to have clear morbidity benefits by certain studies. Nonetheless, future trials are needed to compare the various POC PFTs against gold standard LTA testing and determine their relative impact on mortality and functional outcomes.

## Supplementary Information

**Additional file1: Table S1.** Study quality assessment using Newcastle Ottawa Scale for Cohort Studies and Case–Control Studies. **Table S2** Study quality assessment using JBI Checklist for Case Series.

## Data Availability

Not applicable.

## Abbreviations

CFT: Clot formation time
CT: Clotting time
FFP: Fresh frozen plasma
ICH: Intracranial hemorrhage
INR: International normalized ratio
MCF: Maximum clot firmness
PCC: Prothrombin complex concentrate
PFA: Platelet function analyzer
POC: Point-of-care
PFT: Platelet function test
PRISMA: Preferred Reporting Items for Systematic Reviews and Meta-Analyses
PT: Prothrombin time
PTT: Partial thromboplastin time
ROTEM: Rotational thromboelastography
SCT: Standard coagulation test
TEG: Thromboelastography
