# Supplementary material for: Point-of-care platelet function testing for guided transfusion in neurosurgical management of intracranial hemorrhage: a systematic review
Source: Eur J Med Res. 2022 Oct 1;27:191. doi: 10.1186/s40001-022-00819-4 (PMC9526957; doi:10.1186/s40001-022-00819-4)
Supplement: Supplementary file 1 — Additional file1: Table S1. Study quality assessment using Newcastle Ottawa Scale for Cohort Studies and Case–Control Studies. Table S2 Study quality assessment using JBI Checklist for Case Series. [file 40001_2022_819_MOESM1_ESM.docx]

# ***Supplementary Table 1: Study quality assessment using Newcastle Ottawa Scale for Cohort Studies and Case-Control Studies***

| **Study** | **Selection (scored out of 4)** | **Comparability (scored out of 2)** | **Outcome (scored out of 3)** | **Total (scored out of 9)** |
| --- | --- | --- | --- | --- |
| Cohort | | | | |
| Li 2021 | 4 | 2 | 3 | 9 |
| Vahtera 2019 | 4 | 2 | 3 | 9 |
| Majmundar 2019 | 3 | 1 | 3 | 7 |
| Ellenberger 2017 | 4 | 2 | 3 | 9 |
| Von der Brelie 2018 | 4 | 2 | 3 | 9 |
| Case Control | | | | |
| Rimaitis 2020 | 2 | 2 | 2 | 6 |

#

# ***Supplementary Table 2: Study quality assessment using JBI Checklist for Case Series***

| **Study** | **Selection (scored out of 5)** | **Outcome (scored out of 5)** | **Total (scored out of 10)** |
| --- | --- | --- | --- |
| Beynon 2013 | 2 | 4 | 6 |
